# Wearable Technology Acceptance in Health Care Based on National Culture Differences: Cross-Country Analysis Between Chinese and Swiss Consumers

**DOI:** 10.2196/18801

**Published:** 2020-10-22

**Authors:** Dong Yang Meier, Petra Barthelmess, Wei Sun, Florian Liberatore

**Affiliations:** 1 International Management Institute School of Management and Law Zurich University of Applied Sciences Winterthur Switzerland; 2 Winterthur Institute of Health Economics School of Management and Law Zurich University of Applied Sciences Winterthur Switzerland

**Keywords:** wearables, health care wearables, wearables acceptance, cross culture, national culture, Chinese, Swiss, moderator, digital health, health technology acceptance, smartwatch

## Abstract

**Background:**

The advancement of wearable devices and growing demand of consumers to monitor their own health have influenced the medical industry. Health care providers, insurers, and global technology companies intend to develop more wearable devices incorporating medical technology and to target consumers worldwide. However, acceptance of these devices varies considerably among consumers of different cultural backgrounds. Consumer willingness to use health care wearables is influenced by multiple factors that are of varying importance in various cultures. However, there is insufficient knowledge of the extent to which social and cultural factors affect wearable technology acceptance in health care.

**Objective:**

The aims of this study were to examine the influential factors on the intention to adopt health care wearables, and the differences in the underlying motives and usage barriers between Chinese and Swiss consumers.

**Methods:**

A new model for acceptance of health care wearables was conceptualized by incorporating predictors of different theories such as technology acceptance, health behavior, and privacy calculus based on an existing framework. To verify the model, a web-based survey in both the Chinese and German languages was conducted in China and Switzerland, resulting in 201 valid Chinese and 110 valid Swiss respondents. A multigroup partial least squares path analysis was applied to the survey data.

**Results:**

Performance expectancy (β=.361, *P*<.001), social influence (β=.475, *P*<.001), and hedonic motivation (β=.111, *P*=.01) all positively affected the behavioral intention of consumers to adopt wearables, whereas effort expectancy, functional congruence, health consciousness, and perceived privacy risk did not demonstrate a significant impact on behavioral intention. The group-specific path coefficients indicated health consciousness (β=.150, *P=*.01) as a factor positively affecting only the behavior intention of the Chinese respondents, whereas the factors affecting only the behavioral intention of the Swiss respondents proved to be effort expectancy (β=.165, *P*=.02) and hedonic motivation (β=.212, *P*=.02). Performance expectancy asserted more of an influence on the behavioral intention of the Swiss (β=.426, *P*<.001) than the Chinese (β=.271, *P*<.001) respondents, whereas social influence had a greater influence on the behavioral intention of the Chinese (β=.321, *P*<.001) than the Swiss (β=.217, *P*=.004) respondents. Overall, the Chinese consumers displayed considerably higher behavioral intention (*P*<.001) than the Swiss. These discrepancies are explained by differences in national culture.

**Conclusions:**

This is one of the first studies to investigate consumers’ intention to adopt wearables from a cross-cultural perspective. This provides a theoretical and methodological foundation for future research, as well as practical implications for global vendors and insurers developing and promoting health care wearables with appropriate features in different countries. The testimonials and support by physicians, evidence of measurement accuracy, and easy handling of health care wearables would be useful in promoting the acceptance of wearables in Switzerland. The opinions of in-group members, involvement of employers, and multifunctional apps providing credible health care advice and solutions in cooperation with health care institutions would increase acceptance among the Chinese.

## Introduction

### Background

The global market for wearable devices is growing at a remarkable rate [1]. The terms “wearable technology,” “wearable devices,” and “wearables” all refer to electronic technologies or computers incorporated into items of accessories and clothing, which can comfortably be worn on the body and enable users to collect and self-monitor their health vitals [2]. In this paper, we use the word “wearables” to represent the terms “wearable technology” and “wearable devices.” As the number of potential users continues to grow, wearables will have increasing sociological and cultural impacts [2]. Numerous global companies developing wearables in health care aim to target consumers in many countries. Nevertheless, the intention to accept and adopt wearables varies tremendously among consumers of different cultural backgrounds. It is evident that countries that differ greatly regarding their technological development, social structure, and usage habits have different levels of technology acceptance [3].

Some literature on the influential factors of consumers’ acceptance and adoption of health care wearables is available [4-12]. There are also some studies that focused on the differences in these influential factors in various national cultures with respect to technology acceptance [3,13,14]. However, there is a notable research gap concerning variation in the influential factors on consumers’ acceptance of health care wearables from distinct national cultures [5,6,11]. Based on this, the following research questions were formulated: What are the influential factors on consumers’ behavioral intention to adopt health care wearables? How different are the drivers of the behavioral intention to adopt health care wearables of Chinese and Swiss consumers?

The objective of this study was to investigate certain patterns of influential factors on usage intention of health care wearables by means of comparison of essential acceptance motives and usage barriers of Chinese and Swiss consumers. The different perceptions between Chinese and Swiss users were compared and are discussed in view of the differing national cultures of these two countries. Results of this study will have implications for global digital technology providers to develop and market wearables successfully across borders, as well as possible incentives that the insurers might offer with respect to lifestyle changes to enhance people’s health conditions effectively.

### Prior Research

#### Theories and Models of Wearable Technology Acceptance

Technology acceptance is widely recognized as an aspect of understanding the approval, favorable reception, and continued use of newly introduced devices and systems [3]. Davis [15] developed the first technology acceptance model (TAM), in which perceived usefulness and perceived ease of use were shown to be two main factors affecting the attitude of a user toward new technologies. The TAM was subsequently expanded to include more factors influencing users’ acceptance of computer-related technologies. Recent studies have investigated technology acceptance on the part of consumers, particularly in the area of information technology. This is often performed in the context of the model of unified theory of acceptance and use of technology 2 (UTAUT2) introduced by Venkatesh et al [16]. In the UTAUT2 model, intrinsic motivation such as hedonic motivation and two aspects of consumer behavior, namely price value and habit, were added to the previous construct of UTAUT. The acceptance of medical technology intersects with intimate and personal aspects, and is therefore a highly sensitive topic that sets itself apart from the acceptance of information technology in general [3].

By examining the factors influencing mobile health technology acceptance, Rogers’ Protection Motivation Theory (PMT) [17] has been integrated into the health TAM [4]. Since health care wearables collect users’ personal health information on an ongoing basis, concern about data privacy risk increases. An individual’s decision to adopt health care wearable devices would involve an obvious privacy calculus, in which users may consider the tradeoff between perceived benefit and perceived privacy risk [5]. Based on this theory, various researchers have added and abandoned variables according to the characteristics of technology and the targeted user groups to predict a user’s intention to adopt health care wearables. Gao et al [6] developed an integrated framework comprehensively examining wearable technology acceptance in health care (WTAH) by combining the theories described above [18,19]. Gao et al [6] tested this framework through an empirical survey conducted in China with 462 qualified responses (users of health care wearables) and confirmed that the 8 predictors in their WTAH model, namely performance expectancy, hedonic motivation, effort expectancy, functional congruence, self-efficacy, social influence, perceived vulnerability, and perceived severity, positively influence an individual’s intention to adopt health care wearables; perceived privacy risk negatively affected an individual’s intention to adopt health care wearables. Among all factors, social influence and perceived privacy risk were the most significant predictors [6]. However, this survey was only conducted in China, which did not consider the cultural differences between different countries [6]. Hence, testing whether the approved relationships are still held in other countries is necessary.

#### Influence of National Culture on Technology Acceptance

A country’s cultural values influence technology acceptance [13]. Although the TAM has been used extensively when studying information technology adoption in Western countries, researchers noted that the TAM was not valid when applied to other cultures [13,20]. The UTAUT model was tested in non-Western cultures such as in Saudi Arabia [21], India [22], and China as compared to the United States [23]. These studies provided evidence that there is an interaction between the two phenomena of technology acceptance and national culture.

Several sets of dimensions have been developed to characterize the concept of national culture [24]. At present, at least 6 models of national cultures are widely cited and utilized in the management research literature [14]. These 6 culture models attempt to provide a well-reasoned set of dimensions to facilitate comparison of differing cultures. Among these models, Hofstede and Minkov [25] provided detailed guidelines to explain and measure cultural value differences applied to national culture. To date, these cultural dimensions have been the most commonly used variables for examining models cross-culturally [13,20,24,26]. McCoy et al [27] conducted a simple analysis of variance for each of Hofstede and Minkov’s [25] cultural dimensions measured at the individual level across 8 countries. All of the F-scores obtained were significant at *P*<.001, which provides clear evidence of the existence of national culture (ie, the variance between groups is larger than the variance within groups) [27]. For this reason, this study primarily adopted the Hofstede and Minkov dimensions (hereafter referred to as “Hofstede’s cultural dimensions”) to describe, explain, and to a degree measure the difference in the national cultures of Chinese and Swiss consumers.

Hofstede’s cultural dimensions [28] utilized a national level of analysis, whereas most TAMs were developed for an individual level of analysis. Ford et al [18] stated that it would be beneficial to consider national culture as a moderating variable, since this might play an important role in comparing different populations. Alshare et al [13] confirmed this statement in their empirical study with samples from the United States, Chile, and the United Arab Emirates, showing that national culture dimensions represented by masculinity, power distance, individualism, and uncertainty avoidance moderate four relationships of an extended TAM. McCoy et al [29] showed that high power distance, high masculinity, low uncertainty avoidance, and high collectivism seem to nullify the effects of perceived ease of use or perceived usefulness in the TAM. Taken together, these studies suggested that national culture moderates relationships in the extended TAM, UTAUT, and other related models [13]. Following a similar reasoning, this study examined the moderating role of the national cultures of China and Switzerland with an adapted conceptual model of WTAH.

#### Differences in National Culture Between the Chinese and Swiss

The differences in the national cultures of China and Switzerland according to Hofstede’s country comparison tool [30] are summarized in Table 1. According to Hofstede’s [31] cultural dimension, Switzerland holds the cultural values of high individualism, moderately high uncertainty avoidance, moderately high indulgence, and low power distance. These contrast with the cultural values of the Chinese of low individualism (high collectivism), low indulgence (high restraint), moderately low uncertainty avoidance, and high power distance. Both countries have similarly high values of masculinity and long-term orientation. Multilingualism is an essential part of Switzerland’s identity, with more than 64% of the population speaking German, and approximately 20% speaking French, 8% Italian, and 1% Romansh. Hofstede and Minkov [30] indicated that the German-speaking and French-speaking parts of Switzerland exhibit slightly different cultural values in the aspects of power distance and uncertainty avoidance, but are otherwise very similar with respect to the values of individualism, masculinity, long-term orientation, and indulgence. Therefore, only the cultural values of the German-speaking part of Switzerland were considered for this study.

**Table 1:** Cultural scores of China and Switzerland according to Hofstede and Minkov [30].

Cultural dimensions	China	Switzerland
Power distance	80	34
Individualism	20	68
Masculinity	66	70
Uncertainty avoidance	30	58
Long-term orientation	87	74
Indulgence	24	66

The cultural differences between the Chinese and the Swiss are additionally presented in Table 2, as mapped onto the “Big Five” dimensions in Nardon and Steer’s [14] comparative study of all cultural models. Among these five dimensions, “relationship with the environment” and “time orientation” are additional dimensions that were not explicitly mentioned by Hofstede.

**Table 2:** Country ratings of China and Switzerland in line with “Big Five” dimensions [14].^a^

Cultural dimensions	China	Switzerland
Relationship with the environment	Harmony	Mastery
Social organization	Collectivist+	Individualist
Power distribution	Hierarchical	Egalitarian
Rule orientation	Relationship-based	Rule-based+
Time orientation	Polychronic	Monochronic+

^a^All ratings are comparative, with a “+” sign indicating a stronger tendency toward a particular dimension.

## Methods

### Conceptual Model

**Figure 1:**
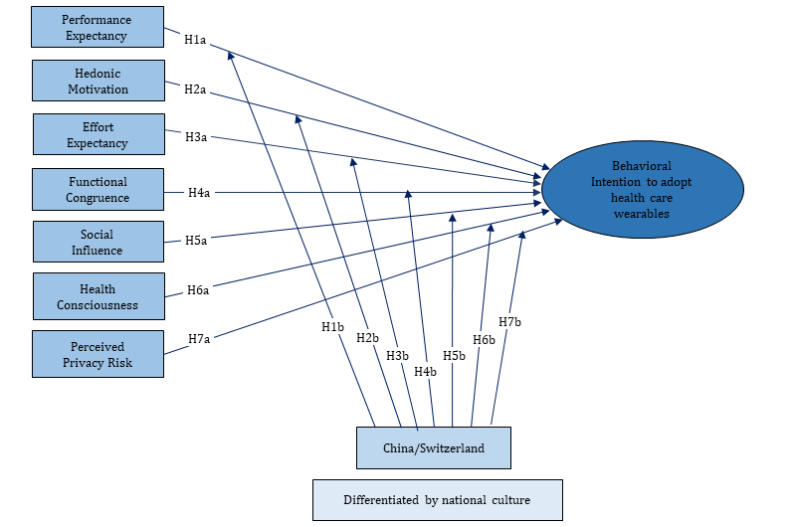
Conceptual model.

To analyze influential factors for the intention to adopt health care wearables, and especially to distinguish the different perspectives between Chinese and Swiss consumers, a conceptual model was developed, which is illustrated in Figure 1. This model was adapted from Gao et al’s [6] WTAH model, since it is one of the most comprehensive models that incorporates consumers’ behavioral intention on technology acceptance (UTAUT2), health behavior (PMT), and privacy calculus theories. The variables such as performance expectancy, hedonic motivation, effort expectancy, functional congruence, social influence, and perceived privacy risk are factors that influence the behavioral intention of consumers using health care wearables, which are adopted from the WTAH model. However, three other variables (self-efficacy, perceived vulnerability, and perceived severity) of the WTAH model based on the PMT were replaced by a single variable, “health consciousness,” in this study, for the following reasons.

Perceived vulnerability refers to the possibility that one will experience a threat of certain diseases, whereas perceived severity represents the extent of the threat of certain diseases [4]. Sun et al [4] argued that the factors relevant to threat appraisal of PMT have only relatively weak (perceived vulnerability) or no (perceived severity) effects on behavioral intention to accept mobile technologies of health services. This is consistent with the meta-analysis results of Floyd et al [4,32]. Part of the health care wearables of focus in this study are consumer-grade devices that are typically used by relatively healthy members of the population who are interested in fitness/wellness. Therefore, the factors in the PMT model would not be suitable for measuring the acceptance of fitness/wellness wearables, which constitute a significant portion of health care wearables. This was especially confirmed during the first round of the pilot study with the first version of the questionnaire, which included the variables of PMT. Some Swiss participants of the pilot study could not answer the related questions, and particularly could not distinguish the question with five different levels of a Likert scale, even when prompted with the situation that they would suffer from a certain disease and have poor knowledge about self-care regarding that disease. Self-efficacy is the belief in one’s ability to use health care wearables to monitor and improve their health condition [6]. The aspects related to this variable are partly covered by the variable effort expectancy, which was remarked as high by the test respondents in the first-round pilot study.

Health consciousness is conceptualized as the extent to which individuals have interest in and are aware of their own health conditions and well-being, and the extent to which a person maintains their own health [7]. A survey by the consulting firm MarketsandMarkets [33] described that the increasing health consciousness among people drives the growth of the wearable technology market. Cho et al [7] as well as Chen and Lin [8] confirmed that health consciousness has a significant direct effect on the perceived ease of use and usefulness of dietary and fitness apps. People’s behavior to maintain their health in the aspect of “health consciousness” covers the aspect of “self-efficacy.” Consequently, this study proposes that health consciousness represents people’s general health concern, awareness, and behavior instead of factors in the PMT.

The definitions of all factors in the proposed model are listed in Table 3, which are adapted from previous published studies [6,7] with minor modifications in wording to fit into the health care wearables context.

**Table 3:** Definitions of factors in the conceptual model.

Construct	Definition/Explanation
Performance expectancy	Degree to which adopting health care wearables will bring effectiveness to users in improving their health condition, which includes monitoring daily physical conditions, making personal health care plans, and reducing health-related threats.
Hedonic motivation	Pleasure or enjoyment derived from adopting and using health care wearables, such as enjoying the technical functions of the devices, sharing data with peers, and feeling of accomplishment after reaching the training goals.
Effort expectancy	Degree of perceived ease of using health care wearables, which includes wearing the device easily on the body, using other devices such as a smartphone to analyze the data, and understanding the data.
Functional congruence	Perceived suitability of health care wearables to fulfill the functional and basic product-related needs such as price reasonability, esthetics, and ergonomic design.
Social influence	Extent to which a user’s decision-making is influenced by others’ perceptions. These “others” include close relationships such as family members and close friends, important people such as employers or peers, professionals such as physicians, and technical specialists.
Health consciousness	Extent to which individuals have interest in and are aware of their own health condition and degree to which health concerns are integrated into their daily activities.
Perceived privacy risk	Perceived risk of reputation damage or other disadvantages by disclosing personal health data to people/organizations unwittingly.
Country China/Switzerland	Country dichotomy of China versus Switzerland distinguished by different national cultural values.
Behavioral intention	Users’ formulation of conscious use or increasing use of health care wearables.

### Hypotheses Related to Influential Factors on Behavioral Intention

The effect of all of the independent variables (except for health consciousness) in this conceptual model were confirmed by Gao et al [6] in their empirical study with Chinese respondents. As both Swiss and Chinese consumers were involved in this study, all of the influential factors in the conceptual model were tested with the entire group of valid respondents to evaluate whether the relationships in this adapted model hold for both groups.

Based on this, the following hypotheses were drawn:

H1a: Performance expectancy is positively correlated with an individual’s intention to adopt health care wearables.

H2a: Hedonic motivation is positively correlated with an individual’s intention to adopt health care wearables.

H3a: Effort expectancy is positively correlated with an individual’s intention to adopt health care wearables.

H4a: Functional congruence is positively correlated with an individual’s intention to adopt health care wearables.

H5a: Social influence is positively correlated with an individual’s intention to adopt health care wearables.

H6a: Health consciousness is positively correlated with an individual’s intention to adopt health care wearables.

H7a: Perceived privacy risk is negatively correlated with an individual’s intention to adopt health care wearables.

### Hypotheses Related to Differences Between the Chinese and Swiss

In the conceptual model, the country (China, Switzerland) distinguished by national culture acts as a moderating variable, which affects the influential degree of the above-mentioned independent variables and the individual’s behavioral intention to adopt the wearables differently.

People in individualist cultures such as the Swiss culture pursue independence and freedom, and therefore advocate self-responsibility and self-reliance [31]. The opinions of close peers would not have much bearing on their decision to adopt wearables. By contrast, wearables enable them to live a more autonomous and freer lifestyle through self-monitoring of their health conditions (eg, not restricted by appointments with a physician, which was confirmed by Swiss interviewees). Therefore, performance expectancy of the Swiss on health care wearables might lead to higher intention to adopt health care wearables. Thus, we hypothesized:

H1b: Performance expectancy has a greater impact on the intention to adopt wearable devices for Swiss consumers than for Chinese consumers.

Swiss respondents exhibited a moderately high value of “indulgence,” which means that they generally place a higher degree of importance on leisure time and having fun [31]. Pleasure or enjoyment derived from using health care wearables, such as enjoying the technical functions of the devices and the feeling of accomplishment after reaching the training goals, might cause the Swiss to have a higher intention to adopt health care variables. Therefore, we hypothesized:

H2b: Hedonic motivation has greater impact on the intention to adopt wearable devices for Swiss consumers than for Chinese consumers.

Ease of use of technology not only influences the user’s motivation but also makes the technology more adaptive in the organization [20]. The individual Swiss consumer tends to solve technological problems by themselves much more than the Chinese consumer, who tends to live in a close community, taking support from the community for granted according to cultural value dimensions [14,20,31]. In addition, the high uncertainty avoidance value causes the Swiss to perceive new technology as more difficult, because the functions and consequences are uncertain [31]. Accordingly, perceived ease of handling health care wearables might have a greater impact on the behavioral intention of Swiss consumers. Therefore, we hypothesized:

H3b: Effort expectancy has greater impact on the intention to adopt wearable devices for Swiss consumers than for Chinese consumers.

The general income in China is dramatically lower than that in Switzerland, and this was clearly reflected in the sampling of this study. Thus, functional and basic product-related needs such as price reasonability, esthetics, and ergonomic design would have more impact on a Chinese consumer’s intention to use health care wearables than a Swiss consumer’s. Therefore, we hypothesized:

H4b: Functional congruence has greater impact on intention to adopt wearable devices of Chinese consumers than for Swiss consumers.

With their generally collectivist values, Chinese consumers are more concerned about the maintenance of group cohesion, put more weight on the opinions of in-group members, and tend to assimilate their opinions or behaviors in their close community [31]. Researchers found that in collectivist countries, the positive effect of social influence on technology acceptance is stronger than that in individualist countries [34]. People in collectivist countries (ie, China) tend to seek out new information from their peers who have already adopted the technology, in contrast to people in individualist countries (ie, Switzerland), who tend to seek information on their own from formal/external sources [20].

The high power distance of Chinese consumers leads to the strong influence of superiors, employers, or authority on adopting wearables for health care or other purposes. For example, some Chinese companies distribute locally produced smartwatches to all of their employees as a kind of fringe benefit for health care. Through the encouragement, support, and influence of the local environment, Chinese consumers find it easy to be in a group of wearable users, which further fosters their intention to adopt the wearables. Therefore, we hypothesized:

H5b: Social influence has greater impact on the intention to adopt wearable devices on the part of Chinese consumers than Swiss consumers.

It is well known that the health care and insurance system in China is far from developed; health care providers are usually overburdened with large volumes of patients and expenses for individuals are relatively high. The health-conscious Chinese might choose to pursue a healthier lifestyle through using wearables to track their health condition and avoid disease rather than going for treatment at a hospital after getting ill. This would save time and expenditures on health care. Therefore, we hypothesized:

H6b: Health consciousness has greater impact on the intention to adopt wearable devices on the part of Chinese consumers than Swiss consumers.

The high uncertainty avoidance of Swiss consumers might make them reluctant to engage with new technology and devices, and to exercise more discretion with personal information. They would perceive the privacy risk much higher than Chinese consumers, and demand clear regulations before adopting digital health care appliances, which might exert more of a negative influence on their intention to use wearables. Therefore, we hypothesized:

H7b: Perceived privacy risk has greater impact on the intention to adopt wearable devices on the part of Swiss consumers than Chinese consumers.

### Construction of Questionnaire

To validate the conceptual model and hypotheses, a quantitative research approach was employed by developing a written questionnaire. Several interviews were first held in Switzerland and China with current users of fitness and medical wearables to ensure the relevance and objectivity of the questions. The measurement items (see Multimedia Appendix 1) for the independent variables performance expectancy, hedonic motivation, effort expectancy, functional congruence, social influence, and perceived privacy risk, and the dependent variable behavioral intention in the questionnaire were adapted from Gao et al [6]; those for health consciousness were adapted from Michaelidou and Hassan [35]. A 5-point Likert scale was employed to measure the items. The questions regarding the cultural value dimension “individualism” were adopted from Hofstede and Minskov’s “Values Survey Module 2013 Questionnaire” [25].

Both variables of “nationality at birth” and “country of residence” were initially assessed to particularly ensure that Chinese living in Switzerland and Swiss living in China were not included in the valid samples, so that the country variable represents a distinct national culture to meet the requirement of reliability. Users and nonusers of health care wearables were included in the collected samples so that the different perceptions and attitudes of both groups (users with high propensity of intention and nonusers with low propensity of intention) were considered.

The questionnaire was translated from English into German and simplified Chinese. The translated versions were corrected by more than two native speakers in each language and have been back-translated. The German language was used for the Swiss questionnaire, as it covers more than 64% of the Swiss population.

Three rounds of pilot studies were conducted; since the measured items are all nonobservable variables, different understandings occur due to language barriers (English, German, and simplified Chinese) and divergent cultural backgrounds. The first pilot study round was conducted initially with 5 Swiss natives representing different age groups. Consequently, the variables of PMT in the WTAH model were replaced by health consciousness. The second and third rounds of pilot studies were conducted with 20 Chinese and 9 Swiss participants, through which the questions were adjusted in both languages a few times to ascertain that all items were formulated clearly, in logical sequences, relevant to the everyday lives of the respondents, and relatively easy to answer.

Smartwatches were selected as the representative health care wearables in this study because they combine the features of consumer and medical-grade devices [9,36]. Most smartwatches can monitor some human physiological signals and biomechanics, and thus act as fitness tracking devices that help users record their daily activities such as automatically recording workout times, tracking heart rates, step counts, and calories burnt [10]. With added apps and sensors, the new generation of smartwatches can further measure heath vitals such as electrocardiography, glucose level, and blood pressure, as well as detect certain diseases such as arrhythmia and seizure [9,37]. Smartwatches are the most frequently purchased wearable devices worldwide currently and will continue to be in the near term [38].

### Data Collection

Both finalized questionnaires were distributed to the Chinese and Swiss populations using web-based survey tools and a snowball sampling method as a convenience sampling technique [39]. The German version, compiled in Survey Monkey, was distributed to Swiss consumers by email, with the request to forward the survey further to their colleagues and friends. The questionnaire was distributed in diverse industry and service companies, fitness centers, leisure and sport clubs, as well as in local communities. The Chinese version, designed in “Wen Juan Xing,” was distributed to Chinese consumers in mainland China by email and the social media platform WeChat with a requirement to share the survey. The collected samples were the responses to the survey from April 2 to April 24, 2019. Until the evening of April 24, 2019, 153 samples were collected from Switzerland and 203 samples were collected from 23 of 32 provinces and municipalities of mainland China (excluding Hong Kong and Macau).

### Data Analysis

First, a descriptive analysis was conducted related to the sample characteristics and the cultural values (individualism/ collectivism) of the Chinese and Swiss. After validity assessments, *t* tests were conducted to compare mean differences in the constructs between the Chinese and Swiss samples. In the last step, the research model was tested using a multigroup partial least squares path analysis method.

## Results

### Sample Description

Among the 356 received responses, 13 incomplete samples that ended in the middle of the questionnaire were deleted, and 32 respondents with other nationalities (2 living in China and 30 in Switzerland) were excluded in the data analysis, being that the moderating factor is a distinct culture of China and Switzerland. This resulted in a complete valid sample of 311 respondents with 201 Chinese respondents living in China and 110 Swiss respondents living in Switzerland. The sample characteristics are displayed in Table 4, showing that the Chinese and Swiss samples represent the population of each country respectively against the current societal and economic backgrounds.

**Table 4:** Sample characteristics (N=311).

Variable		Chinese sample (n=201), n (%)	Swiss sample (n=110^a^), n (%)
**Gender**			
	Male	89 (44.3)	52 (47.3)
	Female	112 (55.7)	58 (52.7)
**Age (years)**			
	16-25	8 (4.0)	10 (9.1)
	26-40	72 (35.8)	33 (30.0)
	41-55	56 (27.9)	33 (30.0)
	56-70	38 (18.9)	29 (26.4)
	>70	27 (13.4)	4 (3.6)
**Monthly income (US $)**			
	<500	16 (8.0)	2 (1.8)
	501-1500	100 (49.8)	4 (3.6)
	1501-3000	36 (17.9)	7 (6.4)
	3001-5000	20 (10.0)	19 (17.3)
	>5001	7 (3.5)	60 (54.5)
	No information	22 (10.9)	17 (15.5)
**Highest education level**			
	Apprenticeship	10 (5.0)	30 (27.3)
	Senior high school	12 (6.0)	5 (4.5)
	College	27 (13.4)	25 (22.7)
	University^b^ and above	143 (71.1)	43 (39.1)
	No information	9 (4.5)	6 (5.5)

^a^One respondent did not answer the questions related to age, monthly income, and education, respectively, in the Swiss sample.

^b^Including universities of applied sciences.

### Difference in Cultural Values of the Chinese and Swiss

Following the 6 cultural dimensions of Hofstede [28], the biggest differentiation between the Chinese and the Swiss exists in the dimension of individualism versus collectivism (opposite of individualism), which was empirically examined in this study. Individualism is the degree to which people in a society are integrated into groups [31]. In a culture with individualistic values (like Switzerland), the ties between individuals are loose; that is, everyone is expected to look after themselves and their immediate family [31]. In cultures with a collectivistic value (like China), people are integrated from birth onward into strong, cohesive in-groups, often extended families that continue protecting them in exchange for unquestioning loyalty [31]. The issue addressed by this dimension is an extremely fundamental one, relevant to all societies in the world [28]. In this study, the question items and calculation methods were based on Hofstede and Minskov’s index formula [25]. The mean value of individualism for the entire Chinese population was 4.00 as compared to that of the Swiss at 46.24. Adding a constant of 20 to the mathematical means results in a value of 24.0 for the Chinese and 66.24 for the Swiss. This conforms almost exactly to Hofstede’s original individualism values (20 vs 68) in Table 1, which empirically confirms the distinguished cultural differences between China (low individualism) and Switzerland (high individualism).

### Validity Assessment

Table 5 depicts the mean values and standard deviations of the constructs shown in Figure 1. This table also includes the reliability and validity statistics. The loadings of all reflective indicators were above .70 and significant, confirming item reliability. In line with this, the values of the composite reliability estimates revealed the internal consistency of the measurement instruments. The reliability estimates for behavioral intention to adopt health care wearables can also be regarded as satisfactory, because Hair et al [40] indicated that the true internal consistency reliability values usually lie between Cronbach α and the composite reliability. In addition, according to the values of the average variance extracted (AVE) statistic, the measurement showed convergent validity.

Based on the quotient of the square root of AVE on the diagonal and the correlation coefficient between constructs in the lines below, according to the Fornell-Larcker criterion [41], the square roots of AVE are larger than the correlation coefficient between constructs; thus, the discriminant validity of the measurement can ultimately be confirmed.

**Table 5:** Descriptive statistics, reliability statistics, and validity statistics.

Variable	Mean (SD)	Cronbach α	Composite reliability	AVE^a^
Performance Expectancy	3.573 (0.806)	.869	0.919	0.791
Hedonic Motivation	3.438 (0.763)	.862	0.915	0.782
Functional Congruence	3.445 (0.678)	.713	0.836	0.631
Effort Expectancy	3.766 (0.803)	.900	0.937	0.833
Social Influence	3.034 (0.990)	.910	0.943	0.848
Health Consciousness	4.028 (0.577)	.806	0.803	0.590
Perceived Privacy Risk	3.393 (0.891)	.848	0.886	0.724
Behavioral Intention	3.129 (1.086)	.927	0.954	0.873

^a^AVE: average variance extracted.

### Differences Between Swiss and Chinese Participants in the Constructs

A *t* test was conducted to compare mean differences between the Chinese and Swiss samples in the constructs of the conceptual model.

As indicated in Table 6, the different responses of Chinese and Swiss respondents toward the variables performance expectancy, functional congruence, effort expectancy, social influence, behavioral intention, and the cultural value of individualism were significant. This indicates that Chinese respondents have higher performance expectancy on the presented wearables than Swiss respondents and are influenced more by their social environment. The moderate significant difference on effort expectancy and functional congruence confirms that the Chinese consider health care wearables easy to use, and they pay more attention to additional functions such as comfort, esthetics, and price value. Differences between Chinese consumers and Swiss consumers toward hedonistic motivation, health consciousness, and perceived privacy risk were not significant. Both groups have quite high health consciousness, perceived privacy risk, and relatively high hedonistic motivation. From the significant differences between Chinese consumers and Swiss consumers in behavioral intention, it can be concluded that the Chinese clearly have more intention to use health care wearables than the Swiss. All of these distinctions are related to the cultural differences between Swiss and Chinese consumers according to cultural value dimensions. 

**Table 6:** Comparing perceptions of the Chinese and Swiss (t test).

Variable	Chinese sample, mean (SD)	Swiss sample, mean (SD)	Mean difference	*P* value
Performance Expectancy	3.74 (0.76)	3.26 (0.79)	0.484	<.001
Hedonic Motivation	3.47 (0.73)	3.38 (0.82)	0.097	.29
Functional Congruence	3.50 (0.70)	3.34 (0.62)	0.168	.03
Effort Expectancy	3.84 (0.77)	3.63 (0.84)	0.215	.03
Social Influence	3.44 (0.84)	2.30 (0.80)	1.136	<.001
Health Consciousness	3.99 (0.61)	4.10 (0.50)	–0.108	.10
Perceived Privacy Risk	3.40 (0.91)	3.38 (0.85)	0.018	.86
Behavioral Intention	3.59 (0.80)	2.29 (1.40)	1.301	<.001
Individualism	4.004 (0.54)	46.24 (61.53)	–42.234	<.001

### Multigroup Partial Least Squares Path Analysis

The group-specific R^2^ value in the Chinese sample was 0.580 and that in the Swiss sample was 0.558, which revealed good explanatory power of the delineated model regarding the behavioral intention to adopt health care wearables.

Table 7 depicts the path coefficients according to the conceptional model (Figure 1). In view of hypotheses H1a-H7a and H1b-H7b, the results showed significant effects of performance expectancy, hedonistic motivation, and social influence on the behavior intention of consumers of health care wearables in general. Performance expectancy, social influence, and health consciousness influenced the behavior intention of the Chinese significantly, whereas performance expectancy, hedonistic motivation, effort expectancy, and social influence had a significant influence on the behavior intention of the Swiss. Group-specific path coefficients showed a stronger influence of performance expectancy on the behavior intention of the Swiss than the Chinese and a stronger influence of social influence on the behavior intention of the Chinese than the Swiss. However, the results of the multigroup analysis indicated that these differences in the path coefficients were not significant when assessed at a level of *P*<.05.

**Table 7:** Group-specific path coefficients for each variable’s influence on behavioral intention and multigroup analysis.

Variable	Path coefficient (*P* value)^a^				*P* value for group differences in path coefficients
	Total sample	Chinese sample	Swiss sample		
Performance Expectancy	0.361 (<.001)	0.271(<.001)	0.426 (<.001)	.17	
Hedonic Motivation	0.111 (.01)	0.082	0.212 (.02)	.28	
Functional Congruence	–0.062	0.142	–0.084	.09	
Effort Expectancy	0.067	–0.003	0.165 (.02)	.08	
Social Influence	0.475 (<.001)	0.321 (<.001)	0.217 (.004)	.31	
Health Consciousness	0.005	0.150 (.01)	–0.042	.08	
Perceived Privacy Risk	–0.042	–0.030	–0.015	.90	

^a^Significance levels are based on a 5000 bootstrap run.

### Principal Results

The results above partially confirmed the proposed hypotheses. Among all of the predictors, performance expectancy, hedonistic motivation, and social influence affected behavioral intention positively, which support hypotheses H1a, H2a, and H5a, respectively. Effort expectancy, functional congruence, health consciousness, and perceived privacy risk did not affect behavior intention significantly, thereby rejecting hypotheses H3a, H4a, H6a, and H7a. Nevertheless, group-specific multigroup analysis with Chinese and Swiss samples indicated that hedonistic motivation and effort expectancy are significant predictors affecting the behavior intention of Swiss consumers positively, but do not affect that of Chinese consumers. By contrast, health consciousness is an important predictor affecting the behavior intention of Chinese consumers positively, but does not appear to have an effect on the behavior intention of Swiss consumers. Performance expectancy is a key factor affecting the behavior intention of both Chinese and Swiss consumers positively, but the degree of influence on Swiss consumers was higher than that on Chinese consumers. Social influence is another key factor affecting the behavior intention of both Chinese and Swiss consumers positively, but the degree of influence on Chinese consumers was higher than that on Swiss consumers. Country variable was not a moderator that differentiated the influence degree of functional congruence or perceived privacy risk toward behavior intention between Chinese and Swiss consumers. These results confirm hypotheses H1b, H2b, H3b, H5b, and H6b, but do not confirm hypotheses H4b and H7b.

## Discussion

### Main Findings

The R^2^ values in the total and subsample analyses indicate that the adapted WTAH model is a suitable conceptual model to assess behavioral intentions to use smartwatches as health care wearables. The multigroup analysis showed no significant differences between the Chinese and Swiss samples with respect to path coefficients. However, clear country-specific differences in the data were still found. First, the bootstrapping results within both samples clearly indicated that the relevance of the factors from the adapted WTAH model in the two sample groups differed. Performance expectancy and social influence appear to play a role in both sample groups to explain behavioral intention, whereas hedonistic motivation and effort expectancy were only key factors for the intention to use a smartwatch for the Swiss sample, and health consciousness only emerged as an important factor affecting behavioral intention for the Chinese sample. Functional congruence did not affect behavior intention in either sample. This could be explained by the fact that functional congruence comprises three items, wearing comfort, fashion, and price reasonability, which belong to three parallel aspects. Although intuitively all of these items relate closely with user intention, their consistency should be further checked.

Moreover, the mean values of perceived privacy risk were relatively high for both Chinese and Swiss consumers, but this was not validated as a significant predictor of the disinclination for consumers to use health care wearables, for consumers in general or for the specific group of Chinese or Swiss consumers. One reason could be that smartwatches were used as the research object in this study, and consequently the related health data are not considered as critical and strictly confidential by users. Another reason could be that as a result of recent advancements in data privacy legislation, people are more familiar and confident with the data protection issue.

### Role of Cultural Values on Acceptability

These results can be explained by the differences in cultural values or social and health care systems between China and Switzerland, which are summarized in Table 8.

Chinese collectivist values and Swiss individualist values were confirmed empirically in this study. As collectivists, Chinese consumers search for information and support on wearables from people around them, attach more importance to others’ opinions, and adapt to their peers. This explains the significantly higher values of effort expectancy, functional congruence, and social influence for the Chinese consumers in the *t* test. Chinese consumers, holding low uncertainty avoidance and “harmony” values toward their surroundings, normally embrace new technology and believe in its effectiveness, which can explain their higher performance expectancy value toward wearables than that of Swiss consumers.

**Table 8:** Influential cultural values on differences between China (CN) and Switzerland (CH).

Variables	Perceptions comparison (*t* test)	Influence degree of country (group- specific path coefficients)	Explanatory cultural dimensions/social systems
Performance expectancy	CN > CH	CN < CH	CN: low IDV^a^; low UAI^b^; “Harmony” CH: high IDV; high UAI; “Mastery”
Hedonistic motivation	No difference	CN ns^c^ CH sig^d^	CN: low IDV; low IND^e^ CH: high IDV; high IND
Effort expectancy	CN > CH	CN ns CH sig	CN: low IDV; low UAI CH: high IDV; high UAI
Functional congruence	CN > CH	ns	CN: low IDV; low income CH: high IDV; high income
Social influence	CN > CH	CN > CH	CN: low IDV; low UAI; high PDI^f^ CH: high IDV; high UAI; low PDI
Health consciousness	No difference	CN sig CH ns	CN: lack of developed health care and insurance system CH: importance on sport activities
Perceived privacy risk	No difference	CN ns CH ns	N/A^g^
Behavioral intention	CN > CH	N/A	CN: low IDV; low UAI; “Harmony” CH: high IDV; high UAI; “Mastery”

^a^IDV: individualism.

^b^UAI: uncertainty avoidance.

^c^ns: not significant.

^d^sig: significant.

^e^IND: indulgence.

^f^PDI: power distance.

^g^N/A: not applicable.

Swiss consumers showed significantly lower behavioral intention than Chinese consumers, which can be explained by their high value of uncertainty avoidance and “mastery” relationship with their surroundings (as compared to those of Chinese consumers). Because using wearables reduces social presence, this could accentuate the feeling of uncertainty. As a novel technology, the side effect, functionality, and measurement accuracy are quite uncertain. Swiss consumers in high uncertainty avoidance cultures will be less oriented toward using wearables than Chinese consumers in low uncertainty avoidance cultures [24]. Furthermore, the “mastery” value of Swiss consumers toward their environment/surroundings makes them inclined to stick with their habits and perceived correctness, which prevents them from trying new devices.

Although the mean values of health consciousness were high for both Chinese and Swiss respondents, it was not validated as a predictor that influences consumer intention to use wearables generally. Nevertheless, through group-specific analysis, health consciousness emerged as a significant predictor for behavior intention for the Chinese but not for the Swiss. The reason probably lies in the fact that Swiss consumers already spend more time on sport or wellness activities to enhance their health, and they generally have no further interest or time to invest in studying wearables for health purposes [7]. This is supported by Seiler and Hüttermann [11], who showed that 51% of nonusers (74% of the full sample) in their study expressed no need to adopt fitness wearables. By contrast, health-conscious Chinese consumers prefer to use wearables to help them track their health, which is likely related to the underdeveloped health system in China with limited health care resources for such a large population; thus, Chinese consumers are open to more diverse and preventive possibilities.

### Limitations

Although some hypotheses were validated, and the criteria of objectivity, reliability, and validity were followed during the whole process of research, this study is subject to certain limitations, and the results should be interpreted with caution.

First, some variables of the conceptual model should be reorganized and reconsidered in future research. For example, health consciousness relates to many different concepts from health awareness, ranging from health concern to health activities. It influences the user’s perception and intention of using wearables multilaterally. Health consciousness might be one of the predetermining factors for other variables such as performance expectancy, effort expectancy, and others, as investigated by some studies with controversial results [7,8]. Therefore, health consciousness should be examined in the future in different model structures. The country variable based on national culture was examined in this study as a moderating variable, which affects the relationship between predictors and outcomes. Cultural values influence many aspects (perception, attitude, intention) further to action. Thus, the country/cultural variables might also be predetermining factors for other predictors. In further research, the roles and distinction of cultural variables should be considered carefully and examined in different model structures as well.

Second, the control variables such as gender, age, education, and income might influence people’s response patterns [42], which means that they can interfere with the predictors to affect a user’s intention to adopt wearables. The control variables and user experiences were not considered in this study. The effect of control variables as moderators alone or as covariates with other moderators such as country/culture should be analyzed in the future.

Third, the external validity of the study is limited. A smartwatch was used as an example of health care wearables, which cannot be generalized to all types of medical wearable devices. In addition, the results cannot be generalized to other countries, as the survey was conducted in only China and Switzerland, and the cultural dimensions were only used to explain the difference between Chinese and Swiss consumers theoretically. It makes more sense to apply cultural values directly in the quantitative analysis in future research. For this purpose, effective methods of obtaining qualified scores of cultural values must be further explored.

Finally, a quantitative approach was applied to examine the conceptual model of this study, which could not provide specific information on certain types of users or devices. In future research, expert or focus group interviews could be conducted regarding a certain type of medical-grade wearable to gain more specific information.

### Comparison With Prior Work

This study is among the first to investigate the influential factors on intention to use health care wearables involving samples from two countries with quite different national cultures. Although most items of the conceptual model were adapted from the framework of Gao et al [6], in this study, the three predictors (self-efficacy, perceived vulnerability, and perceived severity) based on the PMT were replaced by a single variable: health consciousness. Thus, the findings cannot be compared with other studies directly.

Nevertheless, this study verifies that performance expectancy and social influence are the most influential factors on people’s intention to accept health care wearables, which is in line with the results of Gao et al [6]. However, perceived privacy risk was not validated as a significant predictor influencing the behavior intention of consumers negatively, which contrasts with the results of Gao et al [6]. The main reason for this difference could be that more users adopting medical-grade devices were included in the samples of Gao et al’s research because fitness/medical devices were examined as a moderator. This is not the case in our study regarding Chinese consumers, with smartwatches used as an example of health care wearables. Most smartwatch users might not be suffering from severe health problems at this stage. Hence, Chinese respondents might not consider revealing their health information as a privacy risk that would prevent them from using wearables in health care.

### Conclusions

This study examined the factors influencing people’s behavioral intention to accept health care wearables, explored the different perceptions and using intention of Chinese and Swiss consumers, and explained the effect of national culture on these differences. These findings have practical implications for global wearables vendors and insurers to develop and promote health care wearables for consumers from various cultural backgrounds.

Performance expectancy and social influence were the most significant predictors that positively influenced consumer intention to adopt health care wearables. This result indicates that consumers are more affected by the perceived effectiveness of health care wearables and by other people’s opinions. Thus, these factors should be given more attention when global companies develop and market health care wearables. For Switzerland, with cultural values of individualism and high uncertainty avoidance, the positive opinions of professionals such as physicians toward wearables and clearly demonstrated measurement accuracy would make Swiss consumers feel more confident with health care wearables. For China, with cultural values of collectivism and high power distance, the opinions of people in their surroundings (eg, peers, family, and friends) and the engagement of the Chinese working unit (eg, employers’ social benefit) would increase the intention of Chinese consumers to adopt wearables.

Effort expectancy was shown to be an important driver for Swiss consumers to adopt health care wearables. Thus, the wearable devices should be easy to handle to attract Swiss consumers. This includes easy wearing of devices, along with easy analysis and interpretation of the data. Health consciousness emerged as an important driver for Chinese consumers in adopting health care wearables. Thus, multifunctional apps providing feasible health care advice and solutions in cooperation with Chinese health care institutions are essential to attract Chinese consumers.

In addition, this study is one of the first to investigate intentions to adopt health care wearables from a cross-cultural perspective. It thus provides a theoretical foundation in terms of a conceptual model and survey methodology for future research in similar contexts with other countries and cultures.

## Appendix

Multimedia Appendix 1 Measurement items.

## Abbreviations

AVE: average variance extracted
PMT: protection motivation theory
TAM: technology acceptance model
UTAUT: unified theory of acceptance and use of technology
UTAUT2: unified theory of acceptance and use of technology 2
WTAH: wearable technology acceptance in health care
